# The Input Signal Step Function (ISSF), a Standard Method to Encode Input Signals in SBML Models with Software Support, Applied to Circadian Clock Models

**DOI:** 10.1177/0748730412451077

**Published:** 2012-08

**Authors:** R.R. Adams, N. Tsorman, K. Stratford, O.E. Akman, S. Gilmore, N. Juty, N. Le Novère, A.J. Millar, A.J. Millar

**Affiliations:** * SynthSys, University of Edinburgh, Edinburgh, UK; † Department of Computational Neurobiology, European Molecular Biology Laboratory, European Bioinformatics Institute, Hinxton, UK; ‡ Edinburgh Parallel Computing Centre, University of Edinburgh, Edinburgh, UK; § Centre for Systems, Dynamics and Control, School of Engineering, Computing and Mathematics, University of Exeter, UK

**Keywords:** drug treatments, circadian rhythms, systems biology, BioModels SED-ML SBO

## Abstract

Time-dependent light input is an important feature of computational models of the circadian clock. However, publicly available models encoded in standard representations such as the Systems Biology Markup Language (SBML) either do not encode this input or use different mechanisms to do so, which hinders reproducibility of published results as well as model reuse. The authors describe here a numerically continuous function suitable for use in SBML for models of circadian rhythms forced by periodic light-dark cycles. The Input Signal Step Function (ISSF) is broadly applicable to encoding experimental manipulations, such as drug treatments, temperature changes, or inducible transgene expression, which may be transient, periodic, or mixed. It is highly configurable and is able to reproduce a wide range of waveforms. The authors have implemented this function in SBML and demonstrated its ability to modify the behavior of publicly available models to accurately reproduce published results. The implementation of ISSF allows standard simulation software to reproduce specialized circadian protocols, such as the phase-response curve. To facilitate the reuse of this function in public models, the authors have developed software to configure its behavior without any specialist knowledge of SBML. A community-standard approach to represent the inputs that entrain circadian clock models could particularly facilitate research in chronobiology.

Circadian models often include terms representing external, periodic light inputs as a function of time, which mediate entrainment of the model. This external input may represent a pulsed light source as might be used in a laboratory experiment or a more smoothly varying curve. Many circadian models are now publicly available in a standard format, the Systems Biology Markup Language (SBML; Hucka et al., 2003), but an examination of the 30 curated SBML circadian clock models from the BioModels database (Le Novère et al., 2006) revealed substantial heterogeneity in the representation of light input. These include implementation of time-dependent light input using various SBML constructs, use of events to switch from one light condition to another at given times, omission of external light input altogether, or inclusion of one fixed light condition only (e.g., constant light).

This lack of a standard representation of light-input functions hampers the reproduction of published simulation experiments. First, some simulation software may not support all the SBML formalisms used to encode these different light inputs, making comparison between models difficult. Second, modelers need to study the light-input function anew for every circadian model they encounter. Third, model databases accumulate redundant model variants that differ only in the light conditions used.

Here, we suggest a solution to these problems—the Input Signal Step Function (ISSF), which is a reusable, flexible SBML function that can introduce environmental inputs into SBML models of circadian clocks. The ISSF is configured by 6 parameters: *offset* (Θ_0_), *amplitude* (Θ_1_), *pulseDuration* (*T*_*P*_), *cyclePeriod* (*T*_*C*_), *rampDuration* (*T*), and *phase* (φ). The time variable *t* is treated as an implicit variable in SBML.

Figure 1 illustrates the range of behaviors the ISSF can generate. Constant light conditions are obtained by setting Θ_1_ = 0 and choosing an appropriate value for Θ_0_. Single pulses of light are administered by setting the cyclePeriod equal to the simulation time and using a negative offset to control the time of the pulse. The number of cycles is determined by the ratio of the cyclePeriod time to the overall simulation time. Finally, the ramp time is used to control the rate of change between “on” and “off” settings. The complete SBML step function is listed in Supplementary Material S1.

**Figure 1.**
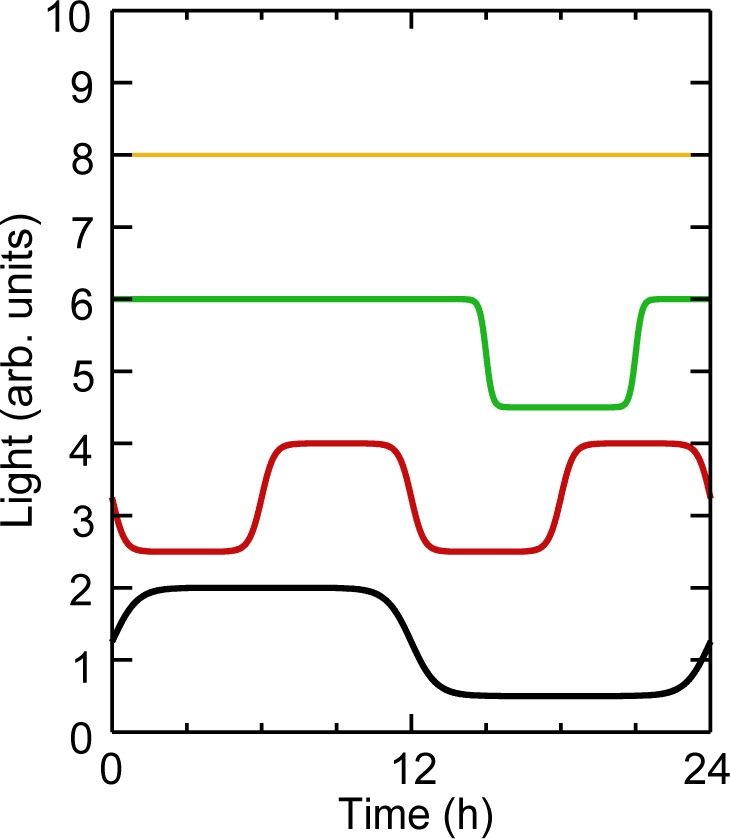
Characterization of ISSF behavior. A number of different time series Θ(*t*) computed from equation (1) that have parameters (from top to bottom): Θ_0_ *=* 9 and Θ_1_ = 0 (i.e., constant light); Θ_0_ = 6.5, Θ_1_ = 1.5, *T*_*C*_ = 24 h, *T*_*P*_ = 3 h, *T* = 0.1 h, and φ = −3 h; Θ_0_ = 4.5, Θ_1_ = 1.5, *T*_*C*_ = 24 h, *T*_*P*_ = 18 h, *T* = 0.25 h, and φ = 3 h; Θ_0_ = 2.5, Θ_1_ = 1.5, *T*_*C*_ = 12 h, *T*_*P*_ *=* 6 h, *T* = 0.5 h, and φ = 6 h; Θ_0_ = 0.5, Θ_1_ = 1.5, *T*_*C*_ = 24 h, *T*_*P*_ = 12 h, *T* = 1 h, and φ = 0 h.

We demonstrated the utility of the ISSF by applying the function to the 3-loop circadian clock model (Locke et al., 2006), publicly available from BioModels (accession BIOMD0000000089). The public SBML model is not able to reproduce the published model’s sensitivity to the photoperiod length because it was encoded in SBML for constant light conditions only. Figure 2 shows that inclusion of the ISSF in this model, parameterizing the photoperiod to be 8 or 16 h, reproduces the published phase shift of *TOC1* mRNA levels. Supplementary Materials S3 directly compare the original and reproduced experiments.

**Figure 2.**
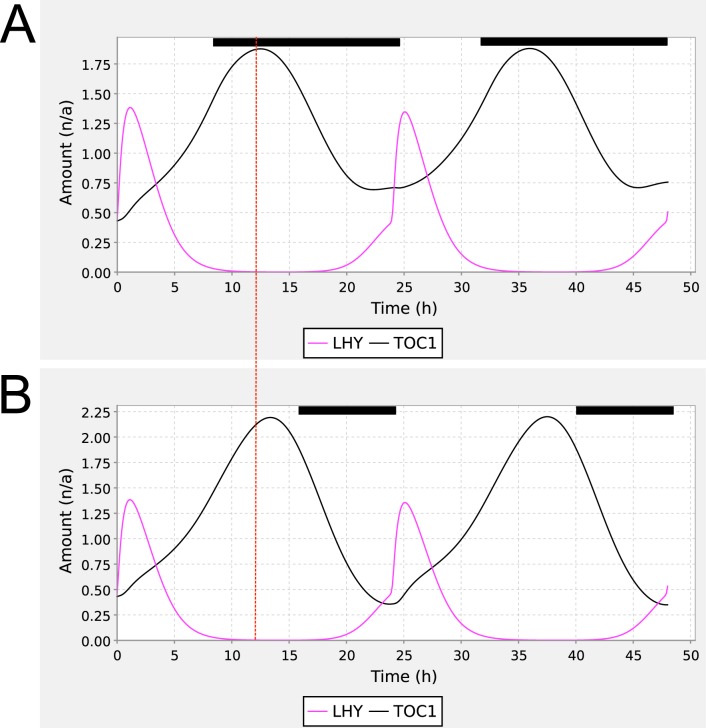
Shift in TOC1 peaks in Locke 3 loop model. Reproduction of Figure 3 from Locke et al. (2006), using the 3-loop circadian clock model BIOMD0000000089 enhanced with the SBML step function. The figure shows simulations of TOC1 mRNA (black) and LHY mRNA (magenta) under photoperiods of (A) LD8:16 and (B) LD16:8. The vertical dotted line highlights the shift in the peak phase of TOC1 mRNA levels from LD8:16 to LD16:8, whereas the peak phase of LHY mRNA is not shifted. Dark periods are indicated using the black bars above the traces. The parameterization of the step function for panel A was Q0 = 0, Q1 = 1, TC = 24 h, TP = 8 h, T = 0.1 h, and f = 0 h (A); for panel B, as for panel A but TP = 16 h.

To help modelers incorporate the ISSF into their models without needing to manipulate SBML directly, we created software to edit the ISSF. Users may configure multiple, independently modifiable, distinct step functions, which may be arbitrarily combined and assigned to a model variable. The software also provides a graphical view of the input functions. Thus, each step function can control multiple parameters, and any parameter can be controlled by multiple step functions.

We used this software to combine 2 step functions to produce an initial LD entrainment phase followed by a 1-h pulse in constant dark, characteristic of the experimental protocol to measure a phase-response curve. We added these functions to a *Neurospora* circadian model (Leloup et al., 1999) and ran a parameter scan experiment in Copasi (Mendes et al., 2009), varying phase to reproduce the behavior whereby a pulse of light when *frq* mRNA levels are low causes a phase advance, while a pulse at high *frq* levels causes phase delay (Figure 3). The ISSF therefore enables specialized circadian protocols to be reproduced by general-purpose software.

**Figure 3.**
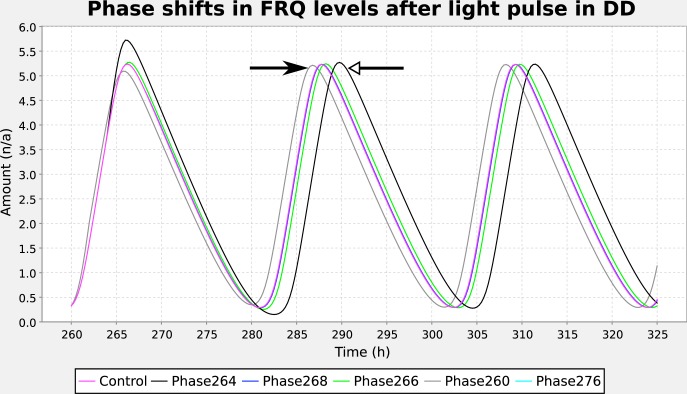
A parameter sweep altering light pulse time can be configured using the ISSF editor. Results of a parameter sweep of the step function’s phase parameter for the Leloup *Neurospora* model (Leloup et al., 1999), reproducing Figure 5 of Leloup et al. (1999). A pulse of light early in the cycle (gray trace, pulse at 260 h, filled arrow) causes phase advance relative to a no–light pulse control (magenta trace), while a pulse of light at 264 h, when the *frq* level is maximal, causes phase delay (black trace, pulse at 264 h, open arrow). Light pulses when *frq* levels are declining do not alter the phase. For the step function controlling the main cycles, 10 cycles of LD (Θ_0_ = 1.6, Θ_1_ = 0.4, *T*_*C*_ = 24 h, *T*_*P*_ = 12 h, *T* = 0.1 h, and ϕ = 0 h) were followed by constant dark (Θ_1_ = 0); for the pulse, Θ_0_ = 0, Θ_1_ = 0.4, *T*_*C*_ = 360 h, *T*_*P*_ = 12 h, *T* = 0.1 h, and ϕ = 0 h, with ϕ ranging from −260 to −276.

To further demonstrate the benefits of standard data formats, we encoded the simulation experiments described above in SED-ML, an XML language to exchange simulation experiment descriptions (Waltemath et al., 2011). By executing these files, it is possible to reproduce the simulation experiments, with no configuration needed by the end-user (Supplementary Materials S2). We also created a new Systems Biology Ontology (Courtot et al., 2011) term to annotate the ISSF (SBO:0000475), facilitating its semantic interpretation.

The general nature of the step function means that it can be applied to any model where periodic forcing of a model parameter is required. We envisage that the function could be applied to other environmental influences, such as temperature, or to periodic experimental perturbations such as drug treatments.

One drawback to this approach is that there is no easy way to incorporate irregular light data as might be recorded in a real laboratory experiment or observation in the natural environment. Such issues are routinely handled by crop science and ecological models, but these lack any model-exchange language equivalent to SBML.

To conclude, we hope that this publication encourages uptake of standard mechanisms for the representation of periodic forcing in SBML models and will motivate other communities to produce standard versions of their core functions.

## Supplementary Material

Supplementary Material

## Appendix

### Development of ISSF in SBML

The mathematical form of the step function follows from the model of Salazar et al. (2009). We consider a light source with constant cycle period *T*_*C*_, which we hold as being in the “on” state for a fixed photoperiod *T*_*P*_ < *T*_*C*_. If we write the “off” state as having light intensity Θ_0_ and the “on” state having intensity Θ_1_, then a function with the desired form is

$$\Theta (t^{\prime })=\Theta_{0}+\frac{1}{2}\Theta_{1}\left\{\left(1+\tanh(t^{\prime }/T)\right)-\left(1+\tanh\left((t^{\prime }-T_{P})/T\right)\right)+\left(1+\tanh\left((t^{\prime }-T_{C})/T\right)\right)\right\}$$

with 0 ≤ *t*′ ≤ *T*_*C*_. We assume that the time variable *t*(*t* ≤ 0) may be reduced to *t*′ via modular arithmetic and, further, an arbitrary phase may be added so that *t*′ = (*t* + φ) modulo *T*_*C*_. This makes later phases more negative, in line with the convention of the circadian community. Finally, the characteristic width of the tanh() function is controlled by *T*. By letting *T* become small compared with any other relevant time scale, a step function can be approached, although *T* = 0 is clearly erroneous. The SBML specification lacks a modulo operation, so the operation *t*′ = (*t* + φ) modulo *T*_*C*_ is implemented using a *floor* function combined with normal arithmetic (e.g., *t*′= *t – floor*(*t*/*T*_*C*_)*T*_*C*_).

The final SBML function is as follows:

$$\begin{array}{l} \Theta_{0}+\\ 0.5*\Theta_{1}*\left\{1.0+\tanh\left(T_{C}*\left(\left(t+\phi \right)/T_{C}-floor\left(\left(t+\phi \right)/T_{C}\right)\right)/T\right)\right\}-\\ 0.5*\Theta_{1}*\left\{1.0+\tanh\left(\left(T_{C}*\left(\left(t+\phi \right)/T_{C}-floor\left(\left(t+\phi \right)/T_{C}\right)\right)-T_{P}\right)/T\right)\right\}+\\ 0.5*\Theta_{1}*\left\{1.0+\tanh\left(\left(T_{C}*\left(\left(t+\phi \right)/TC-floor\left(\left(t+\phi \right)/T_{C}\right)\right)-T_{C}\right)/T\right)\right\} \end{array}$$

### Software Development

The Step Function editor was developed in Java as a plugin for the Systems Biology Software Infrastructure (SBSI, http://www.sbsi.ed.ac.uk), using libSBML (Bornstein et al., 2008). Documentation is available at http://www.sbsi.ed.ac.uk/html/static/visual/index.html#sfplugin. An online ISSF demonstrator (http://www.sbsi.ed.ac.uk/html/mathml/) was developed using JQuery, Ajax, and Java servlets. All simulations were run using COPASI’s LSODA solver (Mendes et al., 2009).

## References

[bibr1-0748730412451077] Bornstein BJ Keating SM Jouraku A Hucka M (2008) LibSBML: An API library for SBML. Bioinformatics 24(6):880-881 18252737 10.1093/bioinformatics/btn051 PMC2517632

[bibr2-0748730412451077] Courtot M Juty N Knüpfer C Waltemath D Zhukova A Dräger A Dumontier M Finney A Golebiewski M Hastings J (2011) Controlled vocabularies and semantics in systems biology. Mol Sys Biol 7:543 10.1038/msb.2011.77 PMC3261705 22027554

[bibr3-0748730412451077] Hucka M Finney A Sauro HM Bolouri H Doyle JC Kitano H Arkin AP Bornstein BJ Bray D Cornish-Bowden A (2003) The Systems Biology Markup Language (SBML): A medium for representation and exchange of biochemical network models. Bioinformatics 19:524-531 12611808 10.1093/bioinformatics/btg015

[bibr4-0748730412451077] Leloup JC Gonze D Goldbeter A (1999) Limit cycle models for circadian rhythms based on transcriptional regulation in *Drosophila* and *Neurospora*. J Biol Rhythms 14(6):433-448 10643740 10.1177/074873099129000948

[bibr5-0748730412451077] Le Novère N Bornstein B Broicher A Courtot M Donizelli M Dharuri H Li L Sauro H Schilstra M Shapiro B (2006) BioModels database: A free, centralized database of curated, published, quantitative kinetic models of biochemical and cellular systems. Nucleic Acids Res 34:D689-D691 16381960 10.1093/nar/gkj092 PMC1347454

[bibr6-0748730412451077] Locke JCW Kozma-Bognár L Gould PD Féher B Kevei E Nagy F Turner MS Hall A Millar AJ (2006) Experimental validation of a predicted feedback loop in the multi-oscillator clock of *Arabidopsis thaliana*. Mol Syst Biol 2:59 17102804 10.1038/msb4100102 PMC1682024

[bibr7-0748730412451077] Mendes P Hoops S Sahle S Gauges R Dada J Kummer U (2009) Computational modeling of biochemical networks using COPASI. Methods Mol Biol 500:17-59 19399433 10.1007/978-1-59745-525-1_2

[bibr8-0748730412451077] Salazar JD Saithong T Brown PE Foreman J Locke JC Halliday KJ Carré IA Rand DA Millar AJ (2009) Prediction of photoperiodic regulators from quantitative gene circuit models. Cell 139(6):1170-1179 20005809 10.1016/j.cell.2009.11.029

[bibr9-0748730412451077] Waltemath D Adams R Bergmann F Hucka M Kolpakov F Miller A Moraru I Nickerson D Sahle S Snoep J (2011) Reproducible computational biology experiments with SED-ML, The Simulation Experiment Description Markup Language. BMC Syst Biol 5(1):198 22172142 10.1186/1752-0509-5-198 PMC3292844

