# Supplementary material for: The Input Signal Step Function (ISSF), a Standard Method to Encode Input Signals in SBML Models with Software Support, Applied to Circadian Clock Models
Source: J Biol Rhythms. 2012 Aug;27(4):328–32. doi: 10.1177/0748730412451077 (PMC3423168; doi:10.1177/0748730412451077)

Supplementary Materials

S1. The SBML step function

```xml
<?xml version="1.0" encoding="utf-8"?>
<functionDefinition id="stepFunction" name="Step function" sboTerm="SBO:0000475">
<math xmlns="http://www.w3.org/1998/Math/MathML">
  <lambda>
    <bvar>
      <ci definitionURL="http://biomodels.net/SBO/#SBO:0000347"> t </ci>
    </bvar>
    <bvar>
      <ci definitionURL="http://biomodels.net/SBO/#SBO:0000256"> offset </ci>
    </bvar>
    <bvar>
      <ci definitionURL="http://biomodels.net/SBO/#SBO:0000381"> amplitude </ci>
    </bvar>
    <bvar>
      <ci definitionURL="http://biomodels.net/SBO/#SBO:0000477"> phase </ci>
    </bvar>
    <bvar>
      <ci definitionURL="http://biomodels.net/SBO/#SBO:0000347"> pulseDuration </ci>
    </bvar>
    <bvar>
      <ci definitionURL="http://biomodels.net/SBO/#SBO:0000476"> cyclePeriod </ci>
    </bvar>
    <bvar>
      <ci definitionURL="http://biomodels.net/SBO/#SBO:0000346"> rampDuration </ci>
    </bvar>
    <apply>
      <plus />
      <apply>
        <minus />
        <apply>
          <plus />
          <ci> offset </ci>
          <apply>
            <times />
            <cn> 0.5 </cn>
            <ci> amplitude </ci>
            <apply>
              <plus />
              <cn> 1 </cn>
              <apply>
                <tanh />
                <apply>
                  <divide />
                  <apply>
                    <times />
                    <ci> cyclePeriod </ci>
                    <apply>
                      <minus />
                      <apply>
                        <divide />
                        <apply>
                          <plus />
                          <ci> t </ci>
                          <ci> phase </ci>
                        </apply>
                        <ci> cyclePeriod </ci>
                      </apply>
                      <apply>
                        <floor />
                        <apply>
                          <divide />
```

```
                      <apply>
                        <plus />
                        <ci> t </ci>
                        <ci> phase </ci>
                      </apply>

                    <ci> cyclePeriod </ci>
                  </apply>
                </apply>
              </apply>
            </apply>
            <ci> rampDuration </ci>
          </apply>
        </apply>
      </apply>
    </apply>
  </apply>
  <apply>
    <times />
    <cn> 0.5 </cn>
    <ci> amplitude </ci>
    <apply>
      <plus />
      <cn> 1 </cn>
      <apply>
        <tanh />
        <apply>
          <divide />
          <apply>
            <minus />
            <apply>
              <times />
              <ci> cyclePeriod </ci>
              <apply>
                <minus />
                <apply>
                  <divide />
                  <apply>
                    <plus />
                    <ci> t </ci>
                    <ci> phase </ci>
                  </apply>
                  <ci> cyclePeriod </ci>
                </apply>
                <apply>
                  <floor />
                  <apply>
                    <divide />
                                                  <apply>
                        <plus />
                        <ci> t </ci>
                        <ci> phase </ci>
                      </apply>
                    <ci> cyclePeriod </ci>
                  </apply>
                </apply>
              </apply>
            </apply>
            <ci> pulseDuration </ci>
          </apply>
          <ci> rampDuration </ci>
        </apply>
      </apply>
    </apply>
  </apply>
</apply>
<apply>
```

```
        <times />
        <cn> 0.5 </cn>
        <ci> amplitude </ci>
        <apply>
          <plus />
          <cn> 1 </cn>
          <apply>
            <tanh />
            <apply>
              <divide />
              <apply>
                <minus />
                <apply>
                  <times />
                  <ci> cyclePeriod </ci>
                  <apply>
                    <minus />
                    <apply>
                      <divide />
                      <apply>
                        <plus />
                        <ci> t </ci>
                        <ci> phase </ci>
                      </apply>
                      <ci> cyclePeriod </ci>
                    </apply>
                    <apply>
                      <floor />
                      <apply>
                        <divide />
                          <apply>
                            <plus />
                            <ci> t </ci>
                            <ci> phase </ci>
                          </apply>
                        <ci> cyclePeriod </ci>
                      </apply>
                    </apply>
                  </apply>
                </apply>
              </apply>
              <ci> cyclePeriod </ci>
            </apply>
            <ci> rampDuration </ci>
          </apply>
        </apply>
      </apply>
    </apply>
  </apply>
 </lambda>
</math>
</functionDefinition>
```

Listing 1: The SBML step function

S2. SED-ML simulation description files

SED-ML is an XML language to describe computational systems biology simulation experiments. We have created self-contained SED-ML resources called SED-ML archives that hold the model and the simulation conditions. These are stored on the PLASMO (http://www.plasmo.ed.ac.uk/) website, and can be simulated directly in any SED-ML compliant software. Full instructions for running these files are given on the Plasmo website. Specifically:

- For Figure 2, visit http://www.plasmo.ed.ac.uk/plasmo/models/model.shtml?accession=PLM_66 and access the SED-ML resources in the 'Supplementary Data Files' section.

- For Figure 3, visit http://www.plasmo.ed.ac.uk/plasmo/models/model.shtml?accession=PLM_51 and access the SED-ML resources in the 'Supplementary Data Files' section.

S3. Direct comparison with previously published results

We made a direct comparison between an SBML version of the published three-loop circadian clock model (Locke et al., 2006) and the StepFunction-enhanced model. The original model uses a sequence of events to switch between light/dark phases. The figure below shows traces for LHY and TOC1, under 8:16 and 16:8 light regimes, for the original model (suffixed '_orig' in the legend) and the StepFunction-enhanced model. The traces for the original and the step function version are virtually identical.

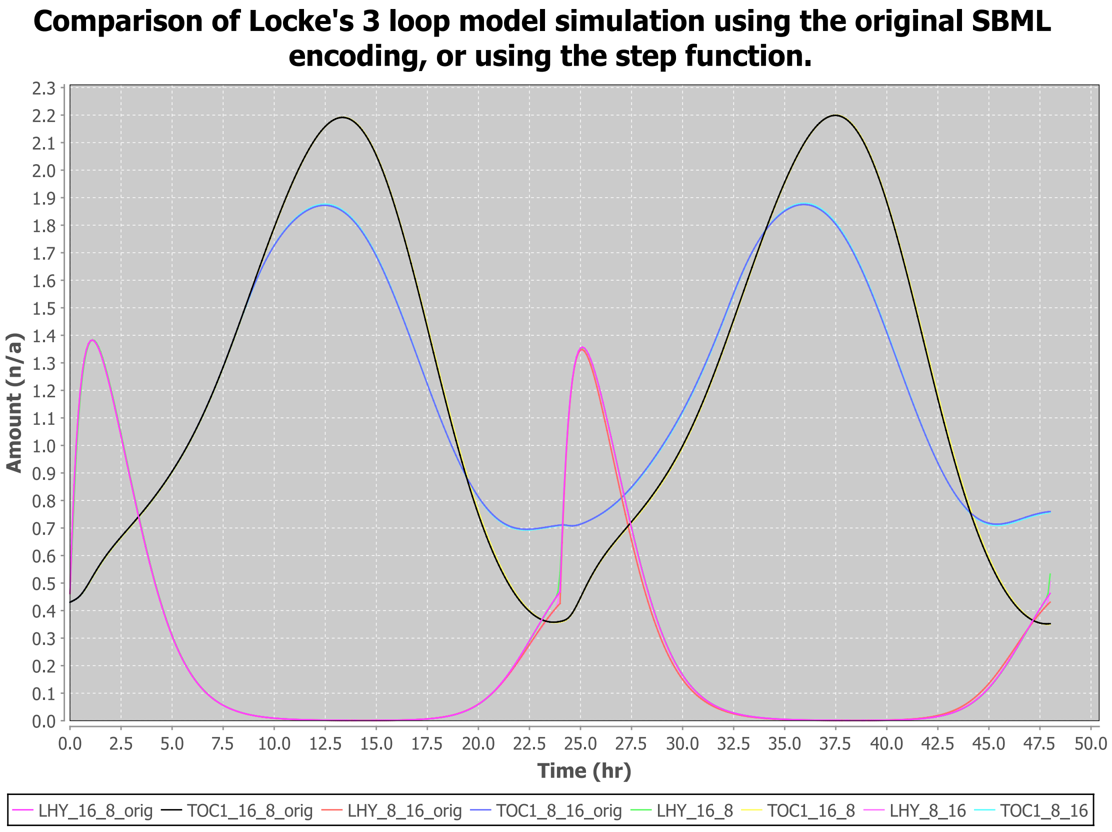

Supplement: Supplementary Material [file DS_10.1177_0748730412451077.pdf]
